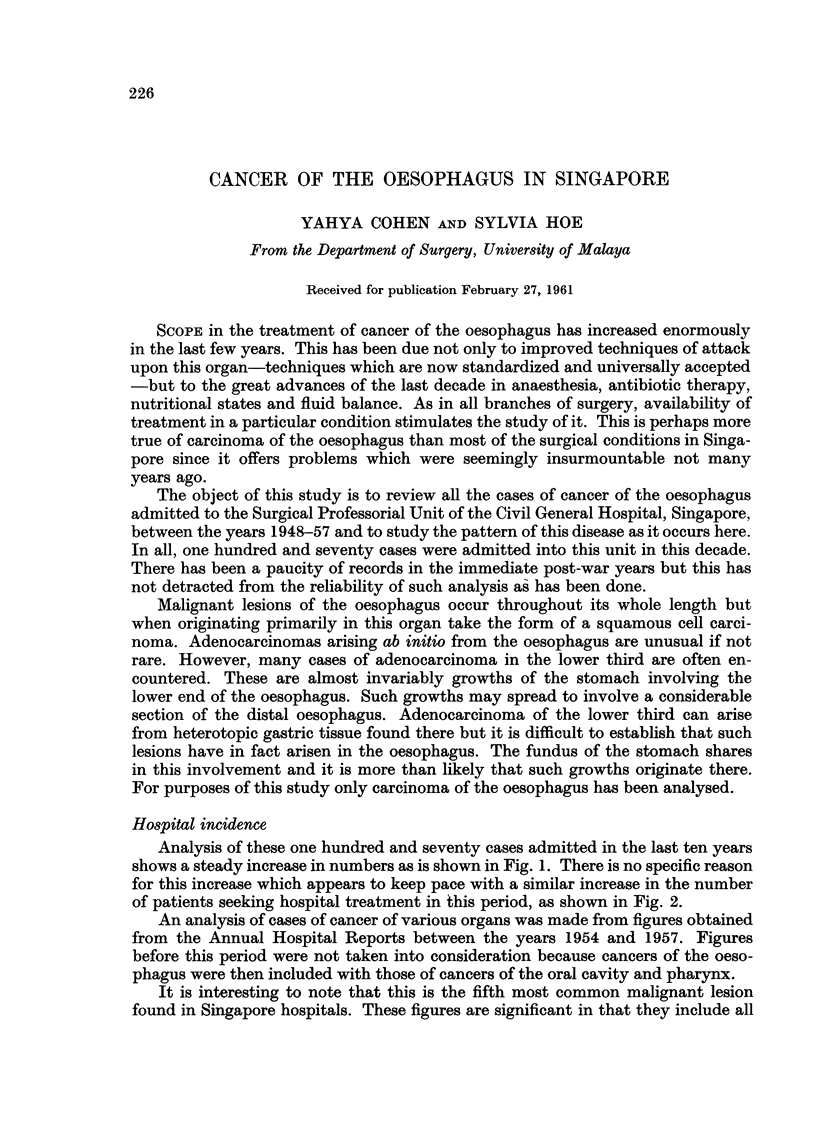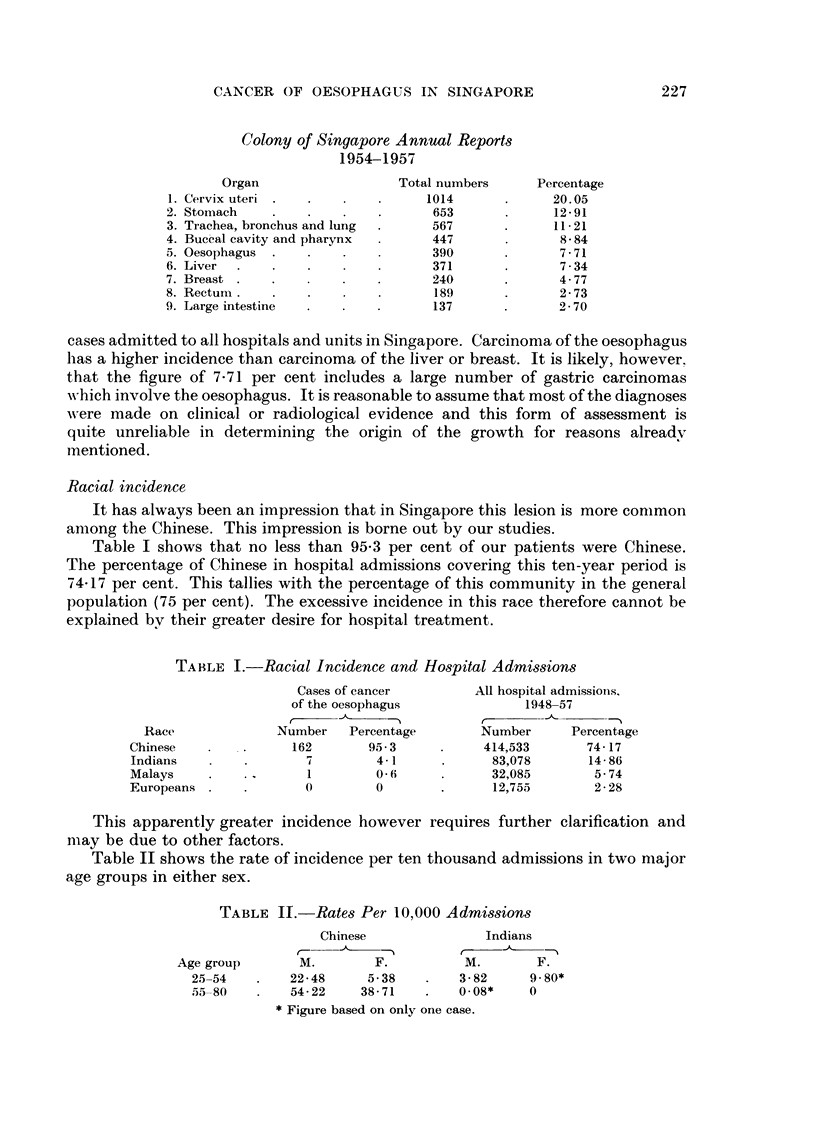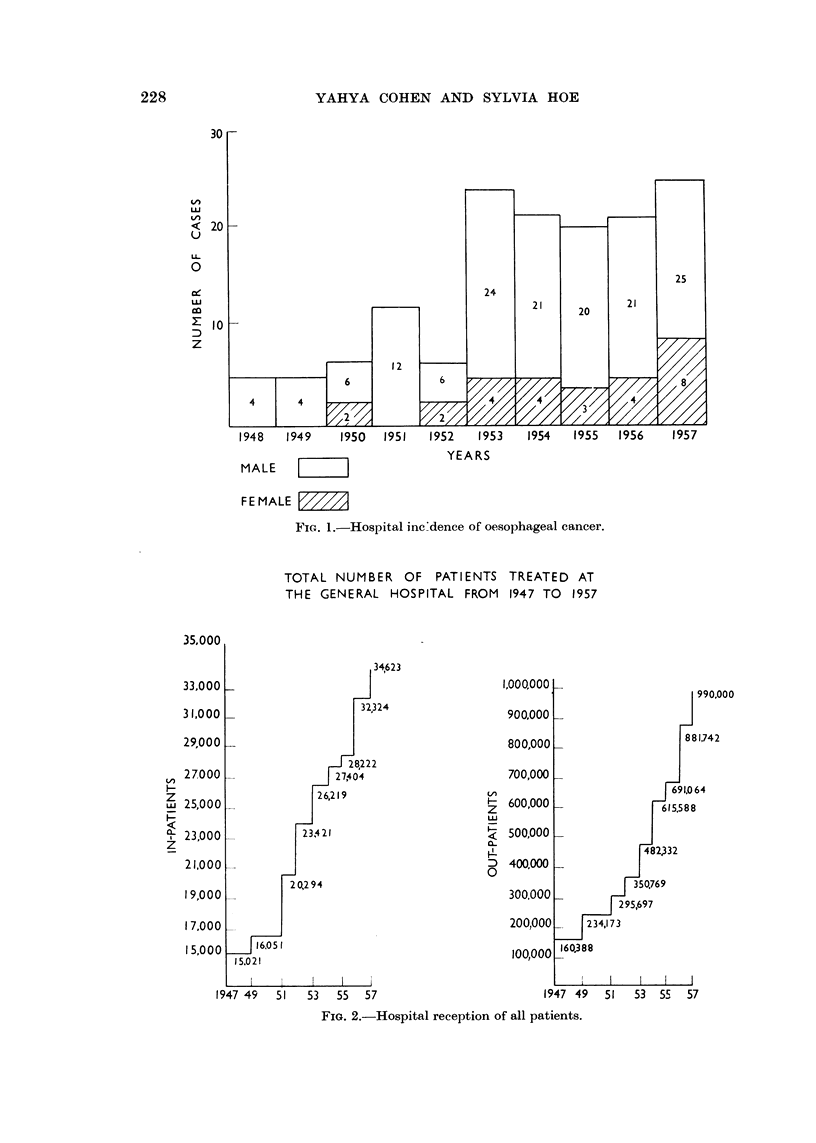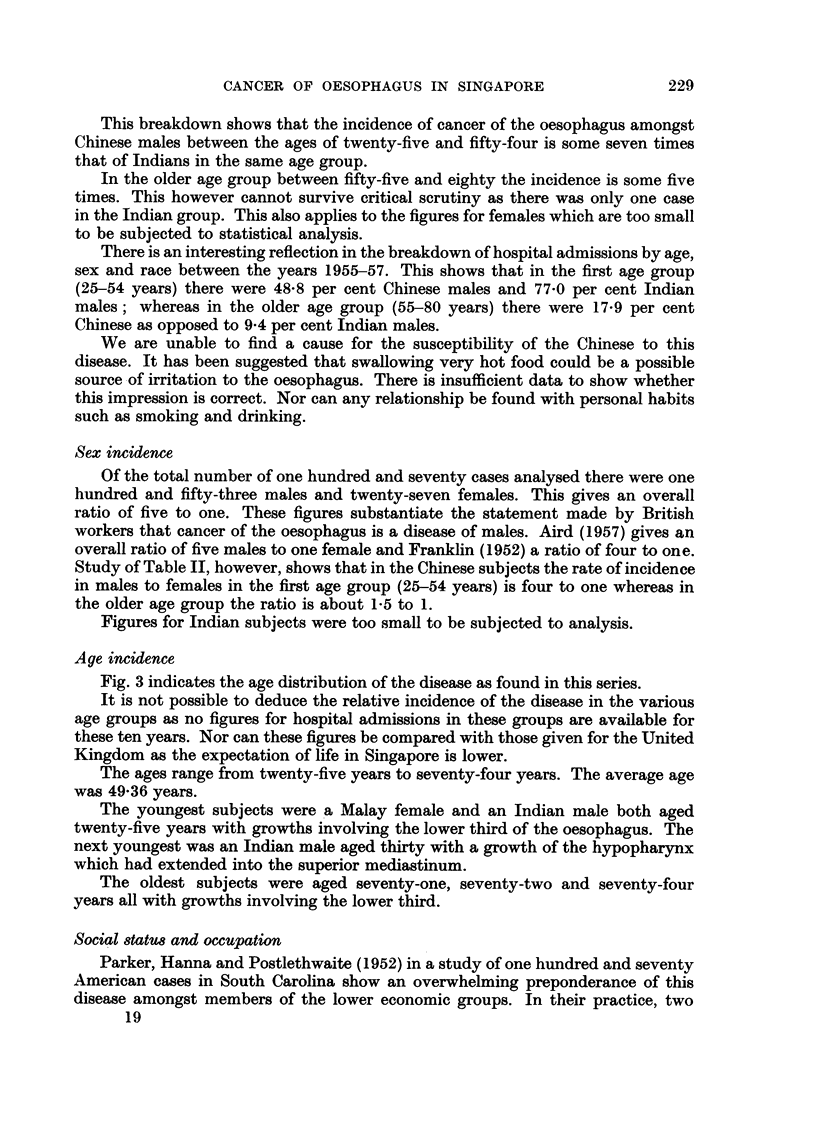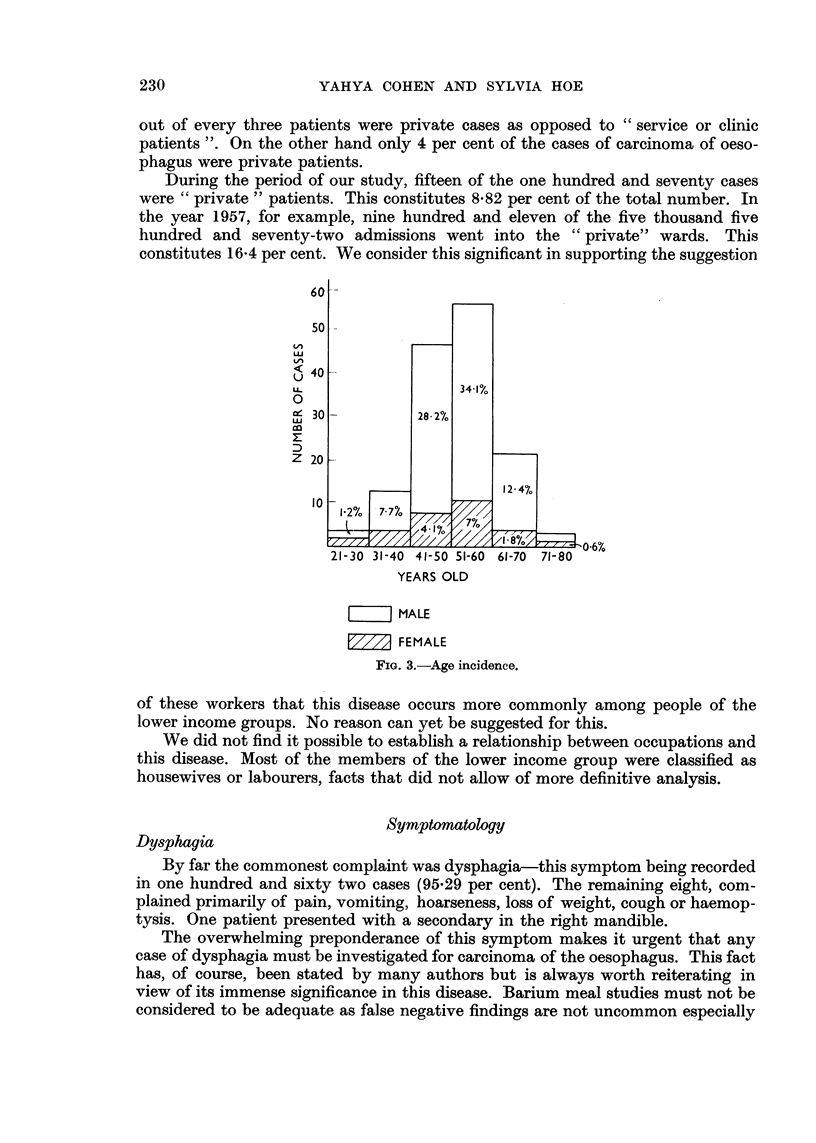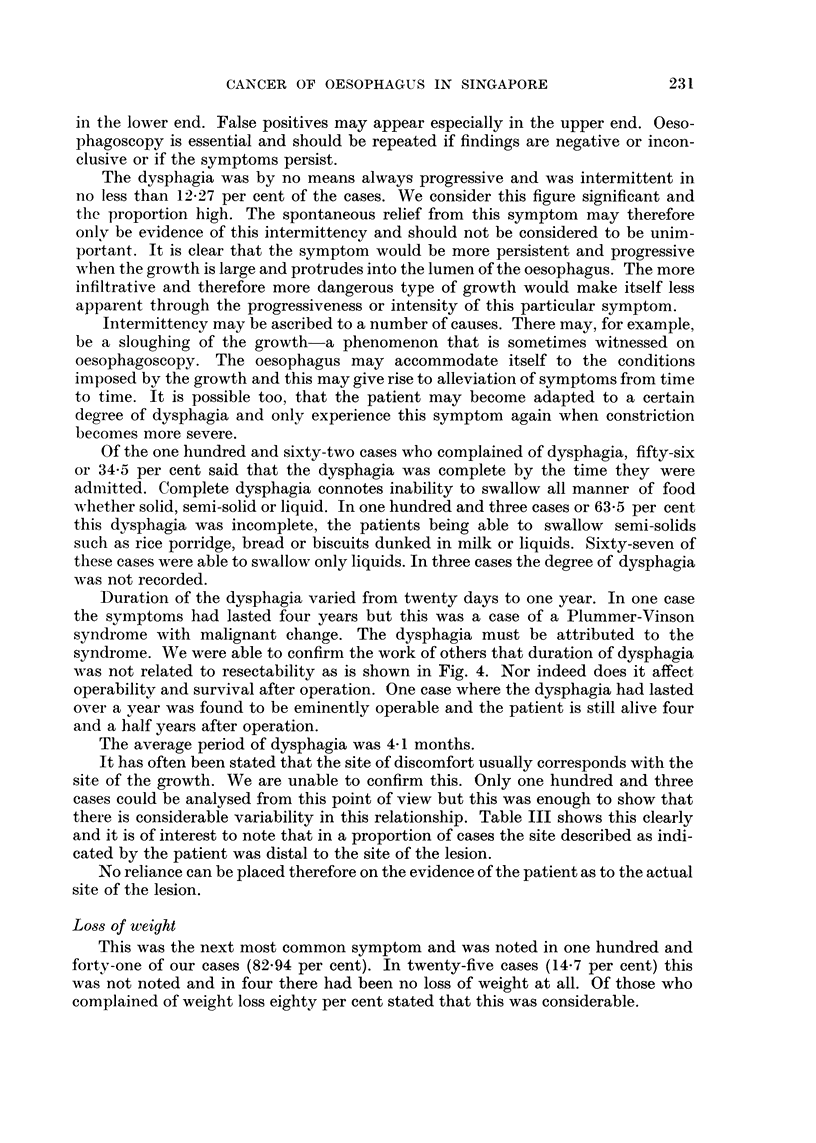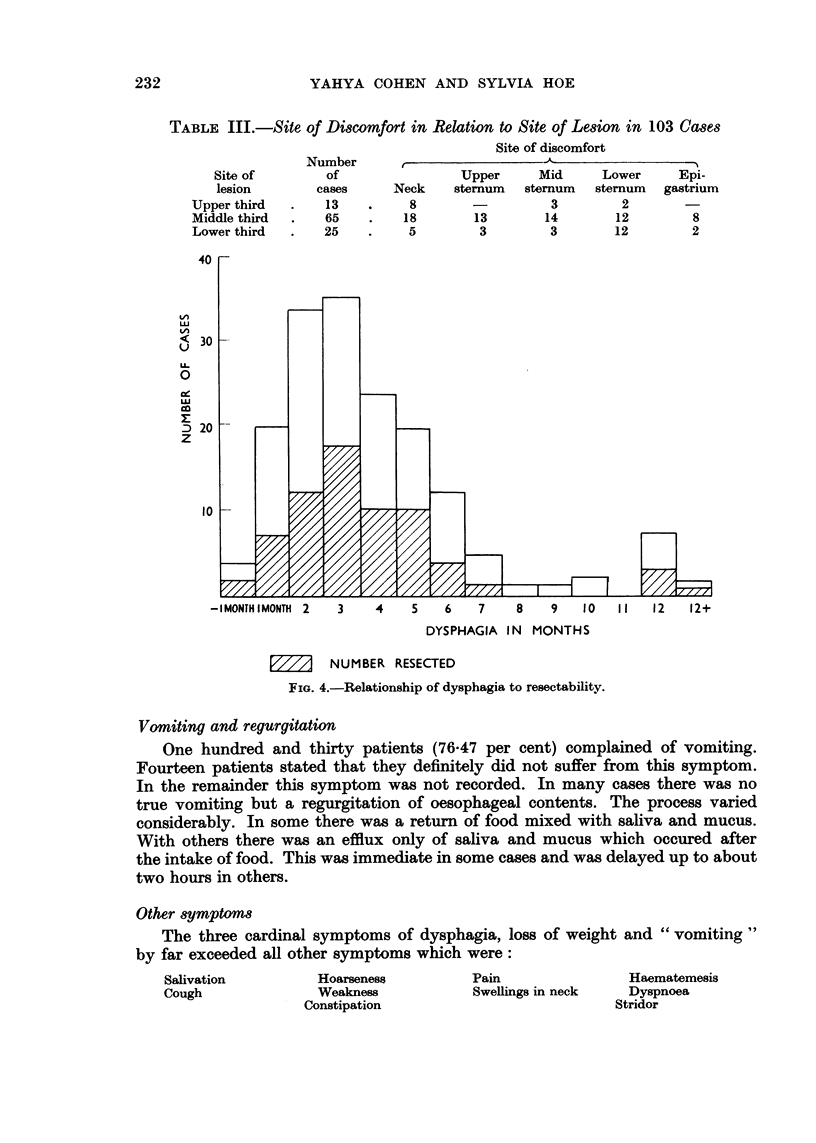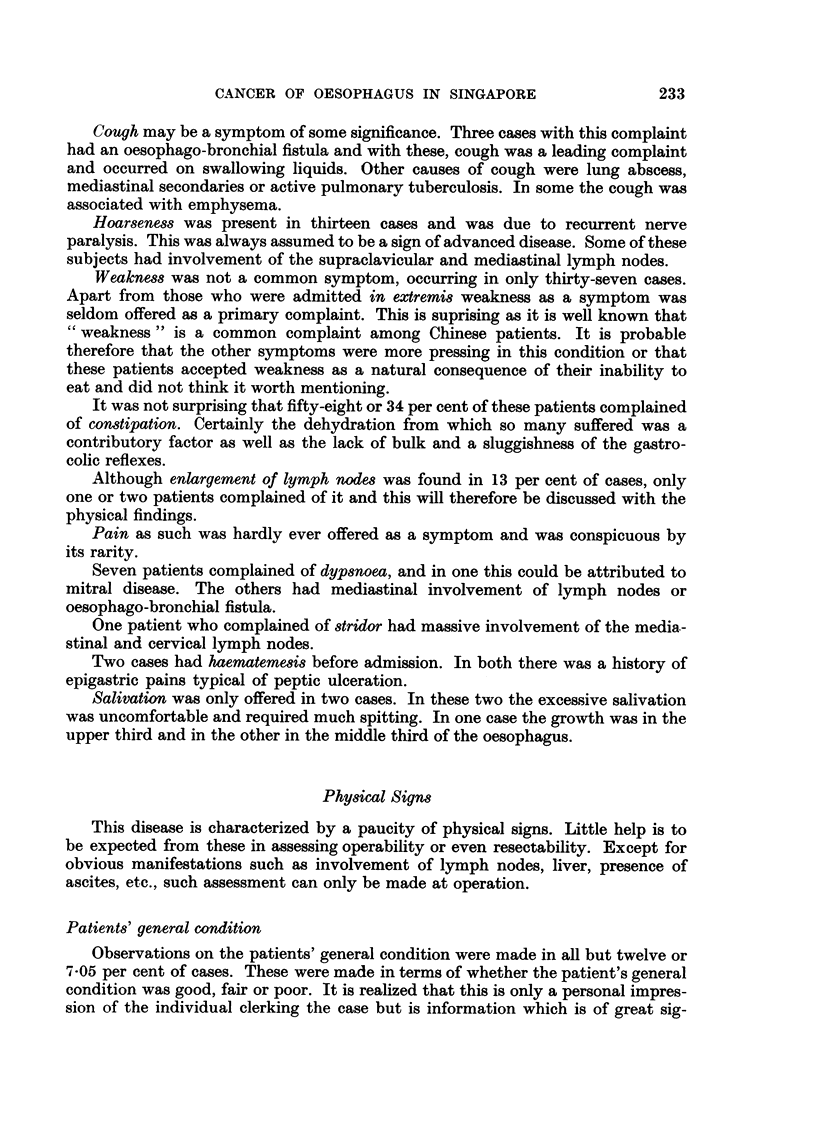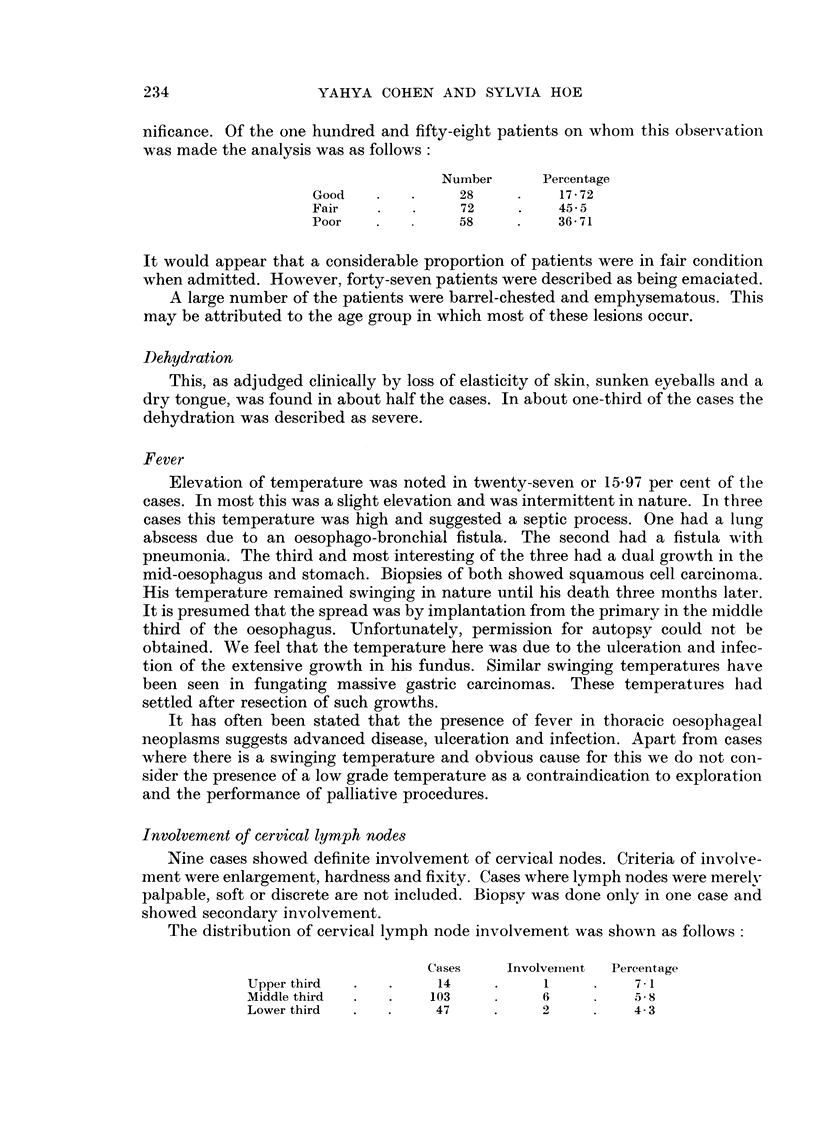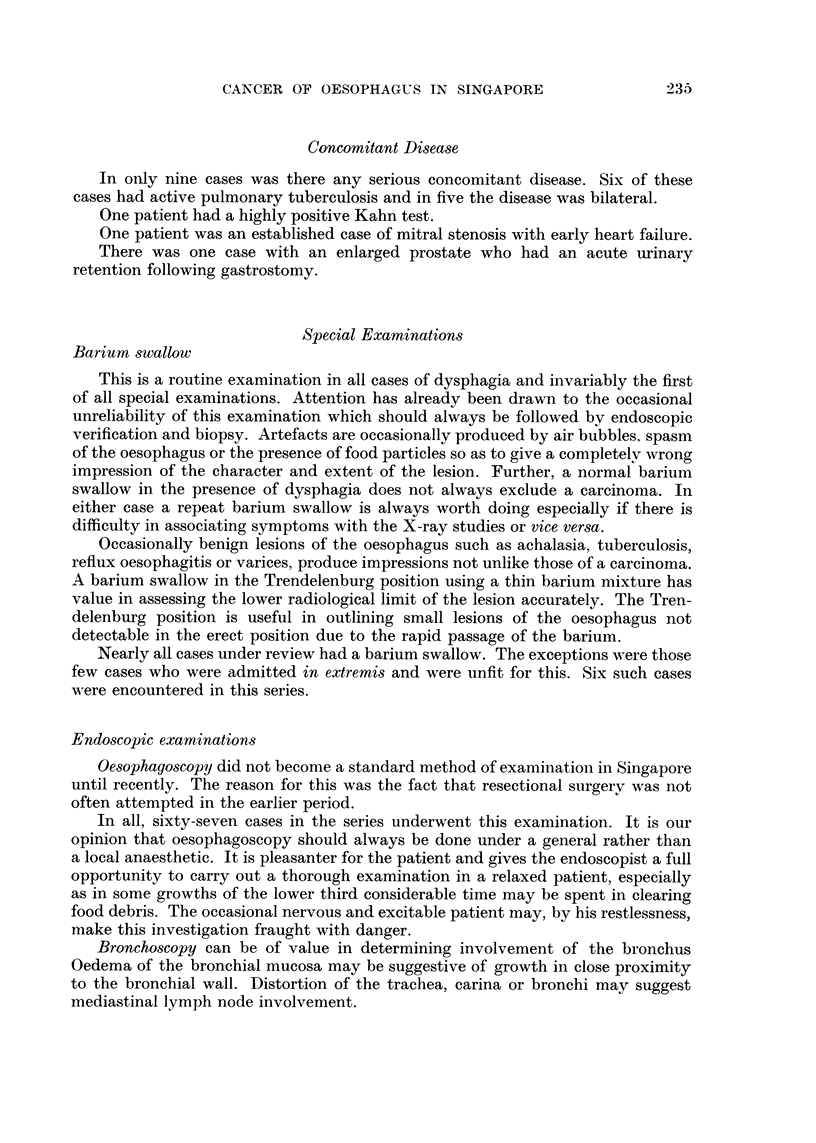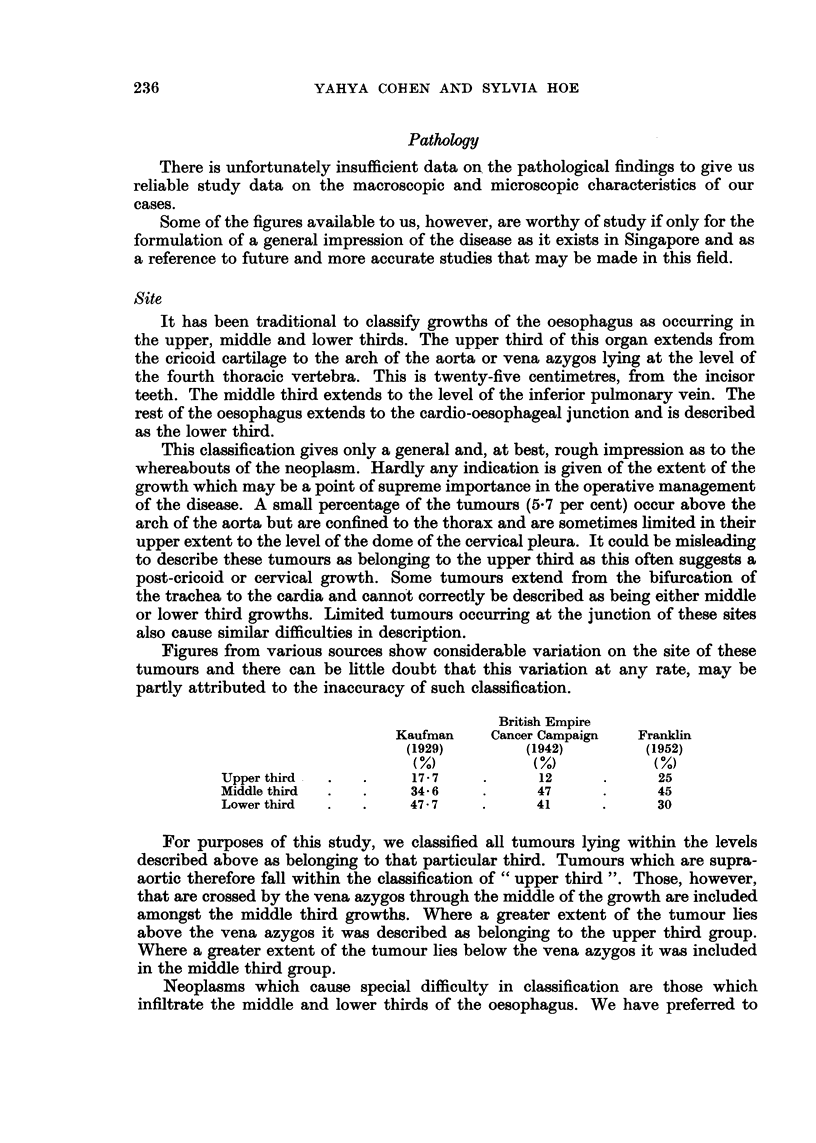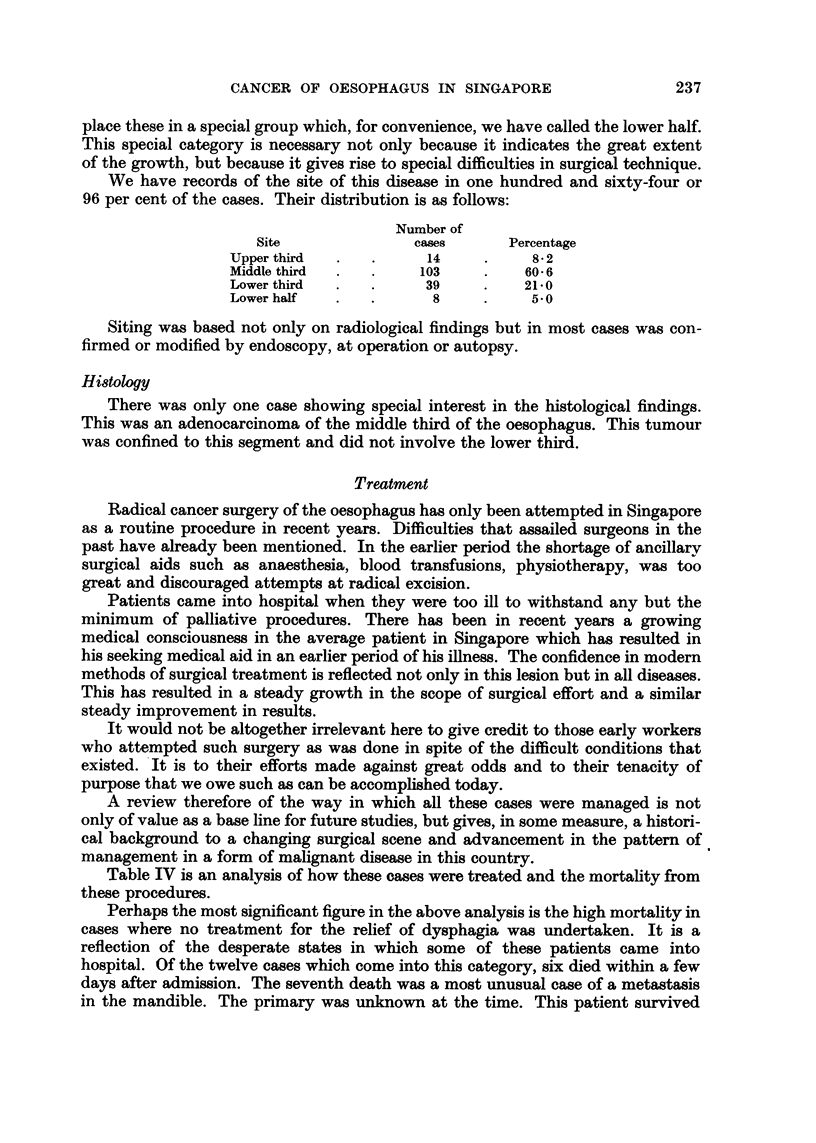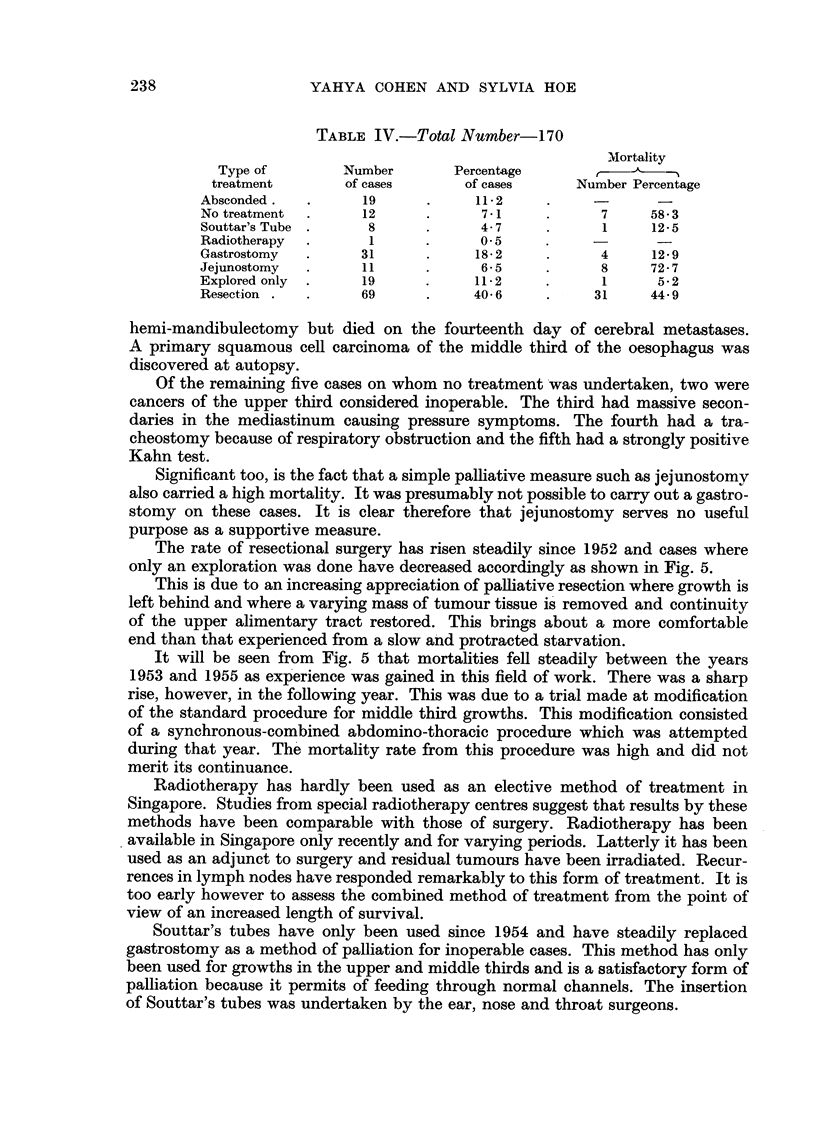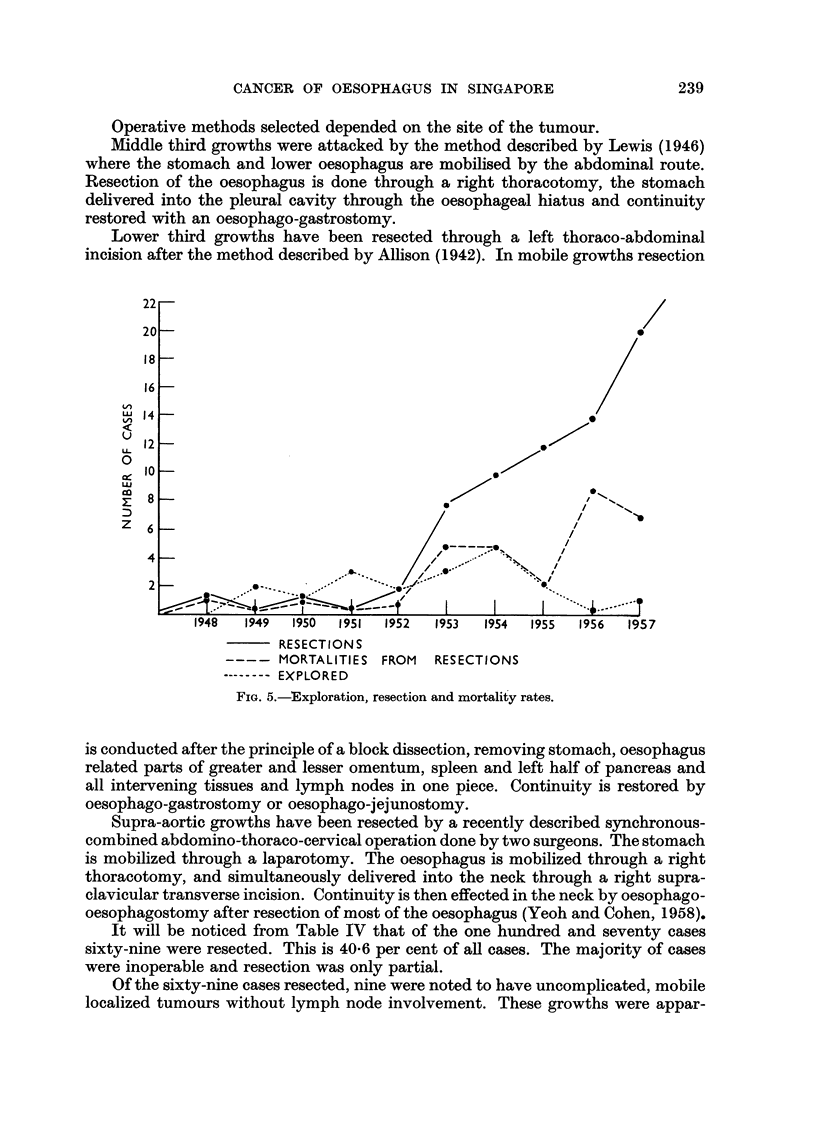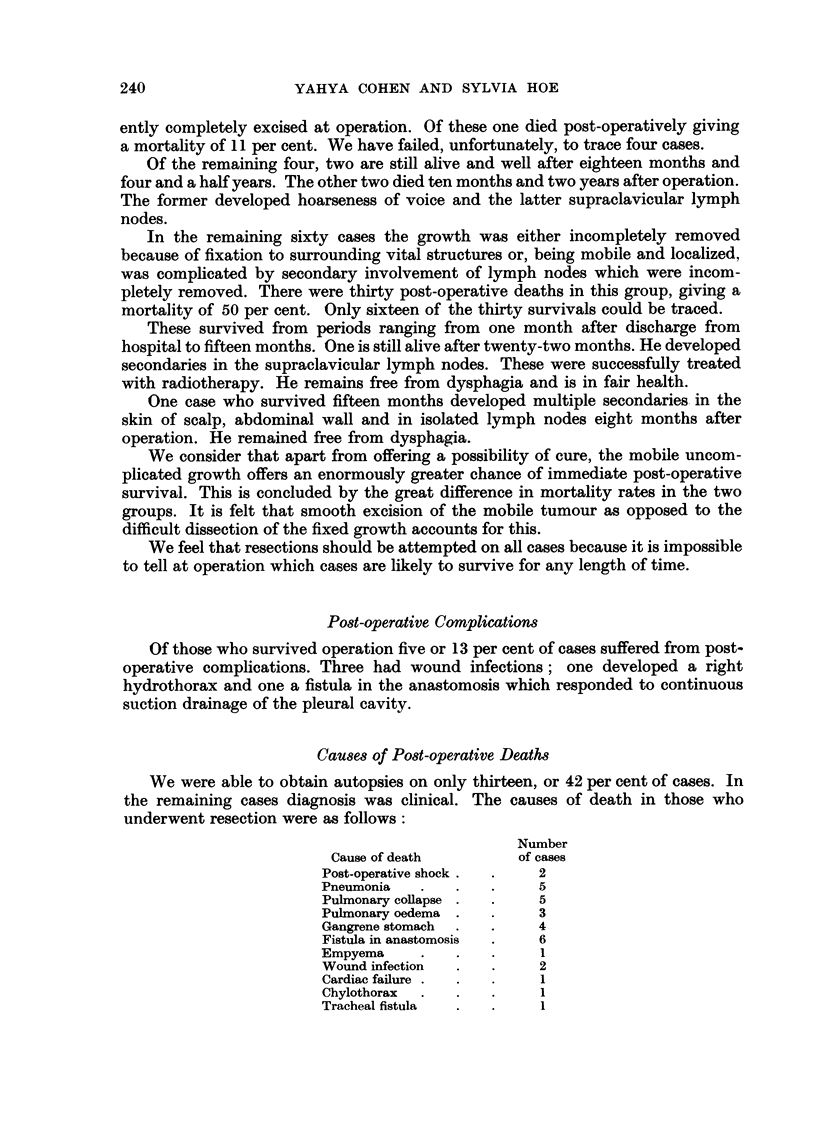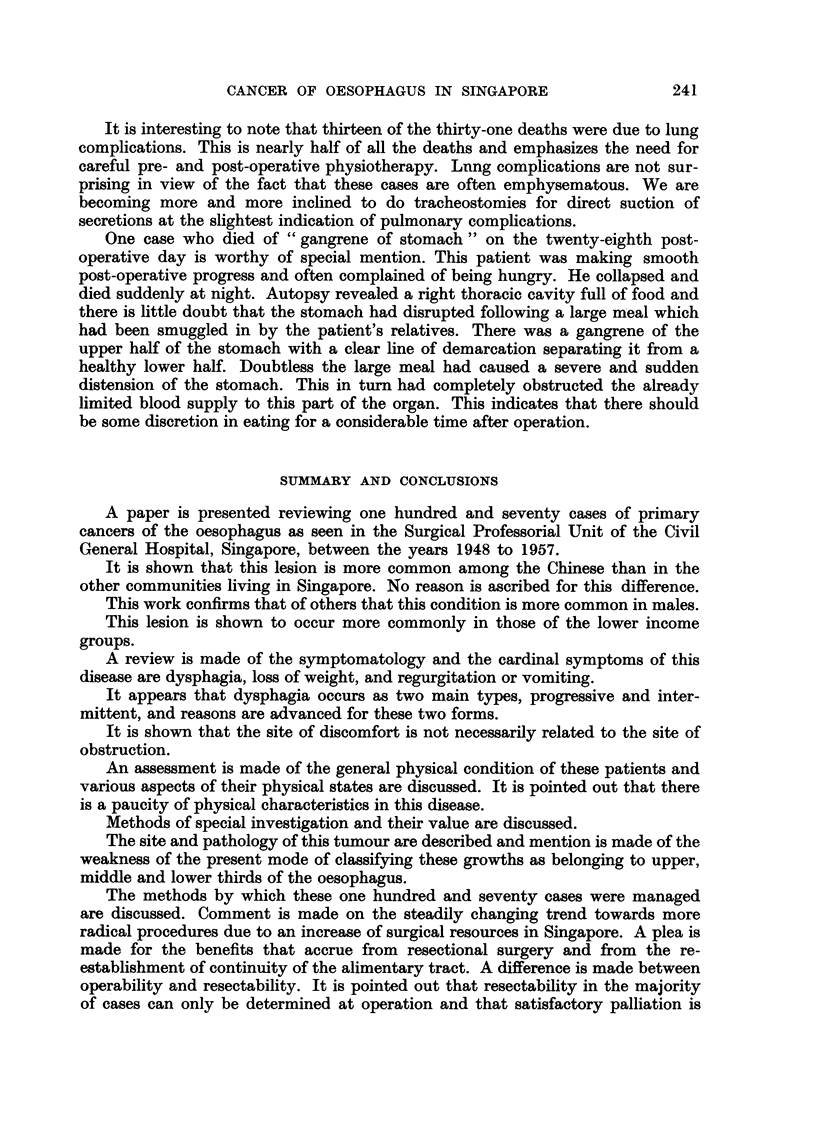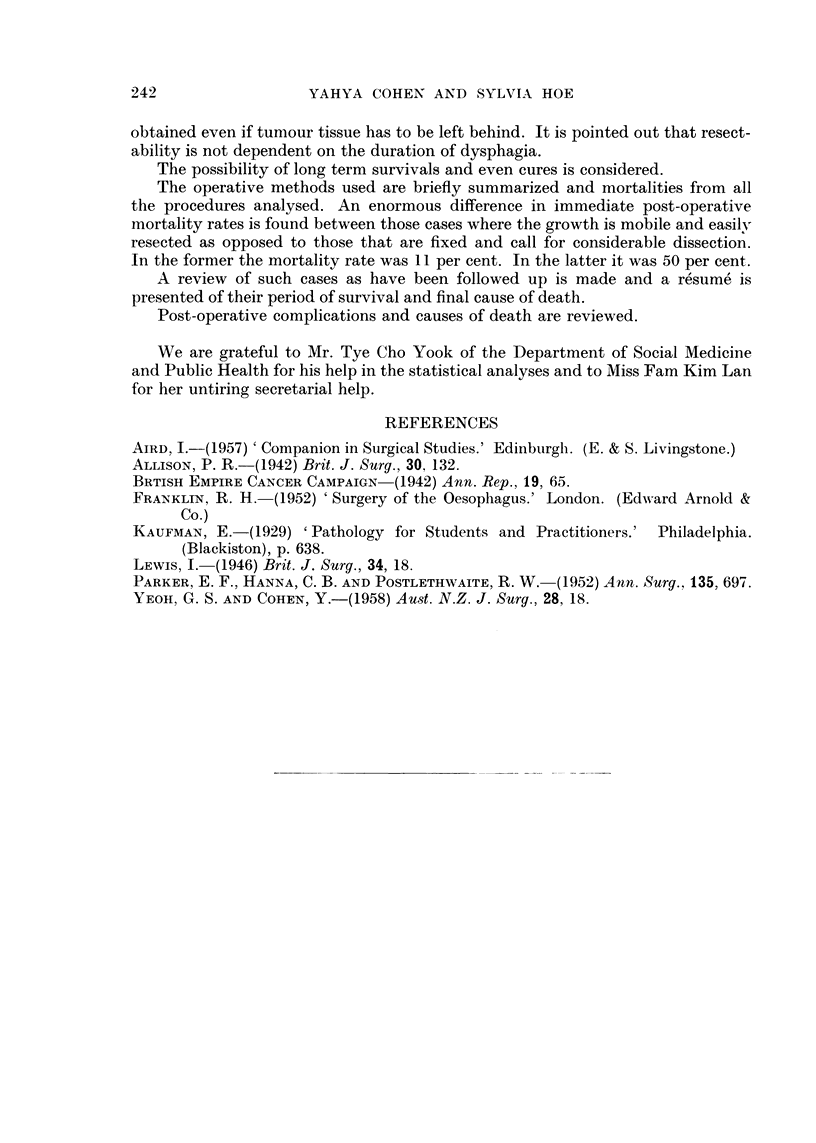# Cancer of the Oesophagus in Singapore

**DOI:** 10.1038/bjc.1961.29

**Published:** 1961-06

**Authors:** Yahya Cohen, Sylvia Hoe


### CANCER OF THE OESOPHAGUS IN SINGAPORE

YAHYA COHEN AND SYLVIA HOE

*From the Department of Surgery, University of Malaya*

Received for publication February 27, 1961

SCOPE in the treatment of cancer of the oesophagus has increased enormously in the last few years. This has been due not only to improved techniques of attack upon this organ—techniques which are now standardized and universally accepted —but to the great advances of the last decade in anaesthesia, antibiotic therapy, nutritional states and fluid balance. As in all branches of surgery, availability of treatment in a particular condition stimulates the study of it. This is perhaps more true of carcinoma of the oesophagus than most of the surgical conditions in Singapore since it offers problems which were seemingly insurmountable not many years ago.

The object of this study is to review all the cases of cancer of the oesophagus admitted to the Surgical Professorial Unit of the Civil General Hospital, Singapore, between the years 1948–57 and to study the pattern of this disease as it occurs here. In all, one hundred and seventy cases were admitted into this unit in this decade. There has been a paucity of records in the immediate post-war years but this has not detracted from the reliability of such analysis as has been done.

Malignant lesions of the oesophagus occur throughout its whole length but when originating primarily in this organ take the form of a squamous cell carcinoma. Adenocarcinomas arising *ab initio* from the oesophagus are unusual if not rare. However, many cases of adenocarcinoma in the lower third are often encountered. These are almost invariably growths of the stomach involving the lower end of the oesophagus. Such growths may spread to involve a considerable section of the distal oesophagus. Adenocarcinoma of the lower third can arise from heterotopic gastric tissue found there but it is difficult to establish that such lesions have in fact arisen in the oesophagus. The fundus of the stomach shares in this involvement and it is more than likely that such growths originate there. For purposes of this study only carcinoma of the oesophagus has been analysed.

*Hospital incidence*

Analysis of these one hundred and seventy cases admitted in the last ten years shows a steady increase in numbers as is shown in Fig. 1. There is no specific reason for this increase which appears to keep pace with a similar increase in the number of patients seeking hospital treatment in this period, as shown in Fig. 2.

An analysis of cases of cancer of various organs was made from figures obtained from the Annual Hospital Reports between the years 1954 and 1957. Figures before this period were not taken into consideration because cancers of the oesophagus were then included with those of cancers of the oral cavity and pharynx.

It is interesting to note that this is the fifth most common malignant lesion found in Singapore hospitals. These figures are significant in that they include all

*Colony of Singapore Annual Reports*
1954–1957

| | Organ | Total numbers | Percentage |
|---|---|---|---|
| 1. | Cervix uteri | 1014 | 20.05 |
| 2. | Stomach | 653 | 12·91 |
| 3. | Trachea, bronchus and lung | 567 | 11·21 |
| 4. | Buccal cavity and pharynx | 447 | 8·84 |
| 5. | Oesophagus | 390 | 7·71 |
| 6. | Liver | 371 | 7·34 |
| 7. | Breast | 240 | 4·77 |
| 8. | Rectum | 189 | 2·73 |
| 9. | Large intestine | 137 | 2·70 |

cases admitted to all hospitals and units in Singapore. Carcinoma of the oesophagus has a higher incidence than carcinoma of the liver or breast. It is likely, however, that the figure of 7·71 per cent includes a large number of gastric carcinomas which involve the oesophagus. It is reasonable to assume that most of the diagnoses were made on clinical or radiological evidence and this form of assessment is quite unreliable in determining the origin of the growth for reasons already mentioned.

*Racial incidence*

It has always been an impression that in Singapore this lesion is more common among the Chinese. This impression is borne out by our studies.

Table I shows that no less than 95·3 per cent of our patients were Chinese. The percentage of Chinese in hospital admissions covering this ten-year period is 74·17 per cent. This tallies with the percentage of this community in the general population (75 per cent). The excessive incidence in this race therefore cannot be explained by their greater desire for hospital treatment.

TABLE I.—*Racial Incidence and Hospital Admissions*

| | Cases of cancer of the oesophagus | | All hospital admissions, 1948–57 | |
|---|---|---|---|---|
| Race | Number | Percentage | Number | Percentage |
| Chinese | 162 | 95·3 | 414,533 | 74·17 |
| Indians | 7 | 4·1 | 83,078 | 14·86 |
| Malays | 1 | 0·6 | 32,085 | 5·74 |
| Europeans | 0 | 0 | 12,755 | 2·28 |

This apparently greater incidence however requires further clarification and may be due to other factors.

Table II shows the rate of incidence per ten thousand admissions in two major age groups in either sex.

TABLE II.—*Rates Per* 10,000 *Admissions*

| | Chinese | | Indians | |
|---|---|---|---|---|
| Age group | M. | F. | M. | F. |
| 25–54 | 22·48 | 5·38 | 3·82 | 9·80* |
| 55–80 | 54·22 | 38·71 | 0·08* | 0 |

* Figure based on only one case.

Fig. 1.—Hospital incidence of oesophageal cancer.

TOTAL NUMBER OF PATIENTS TREATED AT THE GENERAL HOSPITAL FROM 1947 TO 1957

Fig. 2.—Hospital reception of all patients.

This breakdown shows that the incidence of cancer of the oesophagus amongst Chinese males between the ages of twenty-five and fifty-four is some seven times that of Indians in the same age group.

In the older age group between fifty-five and eighty the incidence is some five times. This however cannot survive critical scrutiny as there was only one case in the Indian group. This also applies to the figures for females which are too small to be subjected to statistical analysis.

There is an interesting reflection in the breakdown of hospital admissions by age, sex and race between the years 1955–57. This shows that in the first age group (25–54 years) there were 48·8 per cent Chinese males and 77·0 per cent Indian males ; whereas in the older age group (55–80 years) there were 17·9 per cent Chinese as opposed to 9·4 per cent Indian males.

We are unable to find a cause for the susceptibility of the Chinese to this disease. It has been suggested that swallowing very hot food could be a possible source of irritation to the oesophagus. There is insufficient data to show whether this impression is correct. Nor can any relationship be found with personal habits such as smoking and drinking.

*Sex incidence*

Of the total number of one hundred and seventy cases analysed there were one hundred and fifty-three males and twenty-seven females. This gives an overall ratio of five to one. These figures substantiate the statement made by British workers that cancer of the oesophagus is a disease of males. Aird (1957) gives an overall ratio of five males to one female and Franklin (1952) a ratio of four to one. Study of Table II, however, shows that in the Chinese subjects the rate of incidence in males to females in the first age group (25–54 years) is four to one whereas in the older age group the ratio is about 1·5 to 1.

Figures for Indian subjects were too small to be subjected to analysis.

*Age incidence*

Fig. 3 indicates the age distribution of the disease as found in this series.

It is not possible to deduce the relative incidence of the disease in the various age groups as no figures for hospital admissions in these groups are available for these ten years. Nor can these figures be compared with those given for the United Kingdom as the expectation of life in Singapore is lower.

The ages range from twenty-five years to seventy-four years. The average age was 49·36 years.

The youngest subjects were a Malay female and an Indian male both aged twenty-five years with growths involving the lower third of the oesophagus. The next youngest was an Indian male aged thirty with a growth of the hypopharynx which had extended into the superior mediastinum.

The oldest subjects were aged seventy-one, seventy-two and seventy-four years all with growths involving the lower third.

*Social status and occupation*

Parker, Hanna and Postlethwaite (1952) in a study of one hundred and seventy American cases in South Carolina show an overwhelming preponderance of this disease amongst members of the lower economic groups. In their practice, two out of every three patients were private cases as opposed to " service or clinic patients ". On the other hand only 4 per cent of the cases of carcinoma of oesophagus were private patients.

During the period of our study, fifteen of the one hundred and seventy cases were " private " patients. This constitutes 8·82 per cent of the total number. In the year 1957, for example, nine hundred and eleven of the five thousand five hundred and seventy-two admissions went into the " private" wards. This constitutes 16·4 per cent. We consider this significant in supporting the suggestion

FIG. 3.—Age incidence.

of these workers that this disease occurs more commonly among people of the lower income groups. No reason can yet be suggested for this.

We did not find it possible to establish a relationship between occupations and this disease. Most of the members of the lower income group were classified as housewives or labourers, facts that did not allow of more definitive analysis.

### *Symptomatology*

### *Dysphagia*

By far the commonest complaint was dysphagia—this symptom being recorded in one hundred and sixty two cases (95·29 per cent). The remaining eight, complained primarily of pain, vomiting, hoarseness, loss of weight, cough or haemoptysis. One patient presented with a secondary in the right mandible.

The overwhelming preponderance of this symptom makes it urgent that any case of dysphagia must be investigated for carcinoma of the oesophagus. This fact has, of course, been stated by many authors but is always worth reiterating in view of its immense significance in this disease. Barium meal studies must not be considered to be adequate as false negative findings are not uncommon especially in the lower end. False positives may appear especially in the upper end. Oesophagoscopy is essential and should be repeated if findings are negative or inconclusive or if the symptoms persist.

The dysphagia was by no means always progressive and was intermittent in no less than 12·27 per cent of the cases. We consider this figure significant and the proportion high. The spontaneous relief from this symptom may therefore only be evidence of this intermittency and should not be considered to be unimportant. It is clear that the symptom would be more persistent and progressive when the growth is large and protrudes into the lumen of the oesophagus. The more infiltrative and therefore more dangerous type of growth would make itself less apparent through the progressiveness or intensity of this particular symptom.

Intermittency may be ascribed to a number of causes. There may, for example, be a sloughing of the growth—a phenomenon that is sometimes witnessed on oesophagoscopy. The oesophagus may accommodate itself to the conditions imposed by the growth and this may give rise to alleviation of symptoms from time to time. It is possible too, that the patient may become adapted to a certain degree of dysphagia and only experience this symptom again when constriction becomes more severe.

Of the one hundred and sixty-two cases who complained of dysphagia, fifty-six or 34·5 per cent said that the dysphagia was complete by the time they were admitted. Complete dysphagia connotes inability to swallow all manner of food whether solid, semi-solid or liquid. In one hundred and three cases or 63·5 per cent this dysphagia was incomplete, the patients being able to swallow semi-solids such as rice porridge, bread or biscuits dunked in milk or liquids. Sixty-seven of these cases were able to swallow only liquids. In three cases the degree of dysphagia was not recorded.

Duration of the dysphagia varied from twenty days to one year. In one case the symptoms had lasted four years but this was a case of a Plummer-Vinson syndrome with malignant change. The dysphagia must be attributed to the syndrome. We were able to confirm the work of others that duration of dysphagia was not related to resectability as is shown in Fig. 4. Nor indeed does it affect operability and survival after operation. One case where the dysphagia had lasted over a year was found to be eminently operable and the patient is still alive four and a half years after operation.

The average period of dysphagia was 4·1 months.

It has often been stated that the site of discomfort usually corresponds with the site of the growth. We are unable to confirm this. Only one hundred and three cases could be analysed from this point of view but this was enough to show that there is considerable variability in this relationship. Table III shows this clearly and it is of interest to note that in a proportion of cases the site described as indicated by the patient was distal to the site of the lesion.

No reliance can be placed therefore on the evidence of the patient as to the actual site of the lesion.

*Loss of weight*

This was the next most common symptom and was noted in one hundred and forty-one of our cases (82·94 per cent). In twenty-five cases (14·7 per cent) this was not noted and in four there had been no loss of weight at all. Of those who complained of weight loss eighty per cent stated that this was considerable.

TABLE III.—*Site of Discomfort in Relation to Site of Lesion in* 103 *Cases*

| Site of lesion | Number of cases | Site of discomfort: Neck | Upper sternum | Mid sternum | Lower sternum | Epi-gastrium |
|---|---|---|---|---|---|---|
| Upper third | 13 | 8 | — | 3 | 2 | — |
| Middle third | 65 | 18 | 13 | 14 | 12 | 8 |
| Lower third | 25 | 5 | 3 | 3 | 12 | 2 |

FIG. 4.—Relationship of dysphagia to resectability.

*Vomiting and regurgitation*

One hundred and thirty patients (76·47 per cent) complained of vomiting. Fourteen patients stated that they definitely did not suffer from this symptom. In the remainder this symptom was not recorded. In many cases there was no true vomiting but a regurgitation of oesophageal contents. The process varied considerably. In some there was a return of food mixed with saliva and mucus. With others there was an efflux only of saliva and mucus which occured after the intake of food. This was immediate in some cases and was delayed up to about two hours in others.

*Other symptoms*

The three cardinal symptoms of dysphagia, loss of weight and "vomiting" by far exceeded all other symptoms which were:

| | | | |
|---|---|---|---|
| Salivation | Hoarseness | Pain | Haematemesis |
| Cough | Weakness | Swellings in neck | Dyspnoea |
| | Constipation | | Stridor |

*Cough* may be a symptom of some significance. Three cases with this complaint had an oesophago-bronchial fistula and with these, cough was a leading complaint and occurred on swallowing liquids. Other causes of cough were lung abscess, mediastinal secondaries or active pulmonary tuberculosis. In some the cough was associated with emphysema.

*Hoarseness* was present in thirteen cases and was due to recurrent nerve paralysis. This was always assumed to be a sign of advanced disease. Some of these subjects had involvement of the supraclavicular and mediastinal lymph nodes.

*Weakness* was not a common symptom, occurring in only thirty-seven cases. Apart from those who were admitted *in extremis* weakness as a symptom was seldom offered as a primary complaint. This is suprising as it is well known that "weakness" is a common complaint among Chinese patients. It is probable therefore that the other symptoms were more pressing in this condition or that these patients accepted weakness as a natural consequence of their inability to eat and did not think it worth mentioning.

It was not surprising that fifty-eight or 34 per cent of these patients complained of *constipation*. Certainly the dehydration from which so many suffered was a contributory factor as well as the lack of bulk and a sluggishness of the gastrocolic reflexes.

Although *enlargement of lymph nodes* was found in 13 per cent of cases, only one or two patients complained of it and this will therefore be discussed with the physical findings.

*Pain* as such was hardly ever offered as a symptom and was conspicuous by its rarity.

Seven patients complained of *dypsnoea*, and in one this could be attributed to mitral disease. The others had mediastinal involvement of lymph nodes or oesophago-bronchial fistula.

One patient who complained of *stridor* had massive involvement of the mediastinal and cervical lymph nodes.

Two cases had *haematemesis* before admission. In both there was a history of epigastric pains typical of peptic ulceration.

*Salivation* was only offered in two cases. In these two the excessive salivation was uncomfortable and required much spitting. In one case the growth was in the upper third and in the other in the middle third of the oesophagus.

### *Physical Signs*

This disease is characterized by a paucity of physical signs. Little help is to be expected from these in assessing operability or even resectability. Except for obvious manifestations such as involvement of lymph nodes, liver, presence of ascites, etc., such assessment can only be made at operation.

### *Patients' general condition*

Observations on the patients' general condition were made in all but twelve or 7·05 per cent of cases. These were made in terms of whether the patient's general condition was good, fair or poor. It is realized that this is only a personal impression of the individual clerking the case but is information which is of great significance. Of the one hundred and fifty-eight patients on whom this observation was made the analysis was as follows :

| | Number | Percentage |
|---|---|---|
| Good | 28 | 17·72 |
| Fair | 72 | 45·5 |
| Poor | 58 | 36·71 |

It would appear that a considerable proportion of patients were in fair condition when admitted. However, forty-seven patients were described as being emaciated.

A large number of the patients were barrel-chested and emphysematous. This may be attributed to the age group in which most of these lesions occur.

*Dehydration*

This, as adjudged clinically by loss of elasticity of skin, sunken eyeballs and a dry tongue, was found in about half the cases. In about one-third of the cases the dehydration was described as severe.

*Fever*

Elevation of temperature was noted in twenty-seven or 15·97 per cent of the cases. In most this was a slight elevation and was intermittent in nature. In three cases this temperature was high and suggested a septic process. One had a lung abscess due to an oesophago-bronchial fistula. The second had a fistula with pneumonia. The third and most interesting of the three had a dual growth in the mid-oesophagus and stomach. Biopsies of both showed squamous cell carcinoma. His temperature remained swinging in nature until his death three months later. It is presumed that the spread was by implantation from the primary in the middle third of the oesophagus. Unfortunately, permission for autopsy could not be obtained. We feel that the temperature here was due to the ulceration and infection of the extensive growth in his fundus. Similar swinging temperatures have been seen in fungating massive gastric carcinomas. These temperatures had settled after resection of such growths.

It has often been stated that the presence of fever in thoracic oesophageal neoplasms suggests advanced disease, ulceration and infection. Apart from cases where there is a swinging temperature and obvious cause for this we do not consider the presence of a low grade temperature as a contraindication to exploration and the performance of palliative procedures.

*Involvement of cervical lymph nodes*

Nine cases showed definite involvement of cervical nodes. Criteria of involvement were enlargement, hardness and fixity. Cases where lymph nodes were merely palpable, soft or discrete are not included. Biopsy was done only in one case and showed secondary involvement.

The distribution of cervical lymph node involvement was shown as follows :

| | Cases | Involvement | Percentage |
|---|---|---|---|
| Upper third | 14 | 1 | 7·1 |
| Middle third | 103 | 6 | 5·8 |
| Lower third | 47 | 2 | 4·3 |

### *Concomitant Disease*

In only nine cases was there any serious concomitant disease. Six of these cases had active pulmonary tuberculosis and in five the disease was bilateral.

One patient had a highly positive Kahn test.

One patient was an established case of mitral stenosis with early heart failure.

There was one case with an enlarged prostate who had an acute urinary retention following gastrostomy.

### *Special Examinations*

*Barium swallow*

This is a routine examination in all cases of dysphagia and invariably the first of all special examinations. Attention has already been drawn to the occasional unreliability of this examination which should always be followed by endoscopic verification and biopsy. Artefacts are occasionally produced by air bubbles, spasm of the oesophagus or the presence of food particles so as to give a completely wrong impression of the character and extent of the lesion. Further, a normal barium swallow in the presence of dysphagia does not always exclude a carcinoma. In either case a repeat barium swallow is always worth doing especially if there is difficulty in associating symptoms with the X-ray studies or *vice versa.*

Occasionally benign lesions of the oesophagus such as achalasia, tuberculosis, reflux oesophagitis or varices, produce impressions not unlike those of a carcinoma. A barium swallow in the Trendelenburg position using a thin barium mixture has value in assessing the lower radiological limit of the lesion accurately. The Trendelenburg position is useful in outlining small lesions of the oesophagus not detectable in the erect position due to the rapid passage of the barium.

Nearly all cases under review had a barium swallow. The exceptions were those few cases who were admitted *in extremis* and were unfit for this. Six such cases were encountered in this series.

*Endoscopic examinations*

*Oesophagoscopy* did not become a standard method of examination in Singapore until recently. The reason for this was the fact that resectional surgery was not often attempted in the earlier period.

In all, sixty-seven cases in the series underwent this examination. It is our opinion that oesophagoscopy should always be done under a general rather than a local anaesthetic. It is pleasanter for the patient and gives the endoscopist a full opportunity to carry out a thorough examination in a relaxed patient, especially as in some growths of the lower third considerable time may be spent in clearing food debris. The occasional nervous and excitable patient may, by his restlessness, make this investigation fraught with danger.

*Bronchoscopy* can be of value in determining involvement of the bronchus Oedema of the bronchial mucosa may be suggestive of growth in close proximity to the bronchial wall. Distortion of the trachea, carina or bronchi may suggest mediastinal lymph node involvement.

### *Pathology*

There is unfortunately insufficient data on the pathological findings to give us reliable study data on the macroscopic and microscopic characteristics of our cases.

Some of the figures available to us, however, are worthy of study if only for the formulation of a general impression of the disease as it exists in Singapore and as a reference to future and more accurate studies that may be made in this field.

### *Site*

It has been traditional to classify growths of the oesophagus as occurring in the upper, middle and lower thirds. The upper third of this organ extends from the cricoid cartilage to the arch of the aorta or vena azygos lying at the level of the fourth thoracic vertebra. This is twenty-five centimetres, from the incisor teeth. The middle third extends to the level of the inferior pulmonary vein. The rest of the oesophagus extends to the cardio-oesophageal junction and is described as the lower third.

This classification gives only a general and, at best, rough impression as to the whereabouts of the neoplasm. Hardly any indication is given of the extent of the growth which may be a point of supreme importance in the operative management of the disease. A small percentage of the tumours (5·7 per cent) occur above the arch of the aorta but are confined to the thorax and are sometimes limited in their upper extent to the level of the dome of the cervical pleura. It could be misleading to describe these tumours as belonging to the upper third as this often suggests a post-cricoid or cervical growth. Some tumours extend from the bifurcation of the trachea to the cardia and cannot correctly be described as being either middle or lower third growths. Limited tumours occurring at the junction of these sites also cause similar difficulties in description.

Figures from various sources show considerable variation on the site of these tumours and there can be little doubt that this variation at any rate, may be partly attributed to the inaccuracy of such classification.

| | Kaufman (1929) (%) | British Empire Cancer Campaign (1942) (%) | Franklin (1952) (%) |
|---|---|---|---|
| Upper third | 17·7 | 12 | 25 |
| Middle third | 34·6 | 47 | 45 |
| Lower third | 47·7 | 41 | 30 |

For purposes of this study, we classified all tumours lying within the levels described above as belonging to that particular third. Tumours which are supra-aortic therefore fall within the classification of " upper third ". Those, however, that are crossed by the vena azygos through the middle of the growth are included amongst the middle third growths. Where a greater extent of the tumour lies above the vena azygos it was described as belonging to the upper third group. Where a greater extent of the tumour lies below the vena azygos it was included in the middle third group.

Neoplasms which cause special difficulty in classification are those which infiltrate the middle and lower thirds of the oesophagus. We have preferred to place these in a special group which, for convenience, we have called the lower half. This special category is necessary not only because it indicates the great extent of the growth, but because it gives rise to special difficulties in surgical technique.

We have records of the site of this disease in one hundred and sixty-four or 96 per cent of the cases. Their distribution is as follows:

| Site | Number of cases | Percentage |
|---|---|---|
| Upper third | 14 | 8·2 |
| Middle third | 103 | 60·6 |
| Lower third | 39 | 21·0 |
| Lower half | 8 | 5·0 |

Siting was based not only on radiological findings but in most cases was confirmed or modified by endoscopy, at operation or autopsy.

*Histology*

There was only one case showing special interest in the histological findings. This was an adenocarcinoma of the middle third of the oesophagus. This tumour was confined to this segment and did not involve the lower third.

### *Treatment*

Radical cancer surgery of the oesophagus has only been attempted in Singapore as a routine procedure in recent years. Difficulties that assailed surgeons in the past have already been mentioned. In the earlier period the shortage of ancillary surgical aids such as anaesthesia, blood transfusions, physiotherapy, was too great and discouraged attempts at radical excision.

Patients came into hospital when they were too ill to withstand any but the minimum of palliative procedures. There has been in recent years a growing medical consciousness in the average patient in Singapore which has resulted in his seeking medical aid in an earlier period of his illness. The confidence in modern methods of surgical treatment is reflected not only in this lesion but in all diseases. This has resulted in a steady growth in the scope of surgical effort and a similar steady improvement in results.

It would not be altogether irrelevant here to give credit to those early workers who attempted such surgery as was done in spite of the difficult conditions that existed. It is to their efforts made against great odds and to their tenacity of purpose that we owe such as can be accomplished today.

A review therefore of the way in which all these cases were managed is not only of value as a base line for future studies, but gives, in some measure, a historical background to a changing surgical scene and advancement in the pattern of management in a form of malignant disease in this country.

Table IV is an analysis of how these cases were treated and the mortality from these procedures.

Perhaps the most significant figure in the above analysis is the high mortality in cases where no treatment for the relief of dysphagia was undertaken. It is a reflection of the desperate states in which some of these patients came into hospital. Of the twelve cases which come into this category, six died within a few days after admission. The seventh death was a most unusual case of a metastasis in the mandible. The primary was unknown at the time. This patient survived

TABLE IV.—*Total Number*—170

| Type of treatment | Number of cases | Percentage of cases | Mortality Number | Mortality Percentage |
|---|---|---|---|---|
| Absconded | 19 | 11·2 | — | — |
| No treatment | 12 | 7·1 | 7 | 58·3 |
| Souttar's Tube | 8 | 4·7 | 1 | 12·5 |
| Radiotherapy | 1 | 0·5 | — | — |
| Gastrostomy | 31 | 18·2 | 4 | 12·9 |
| Jejunostomy | 11 | 6·5 | 8 | 72·7 |
| Explored only | 19 | 11·2 | 1 | 5·2 |
| Resection | 69 | 40·6 | 31 | 44·9 |

hemi-mandibulectomy but died on the fourteenth day of cerebral metastases. A primary squamous cell carcinoma of the middle third of the oesophagus was discovered at autopsy.

Of the remaining five cases on whom no treatment was undertaken, two were cancers of the upper third considered inoperable. The third had massive secondaries in the mediastinum causing pressure symptoms. The fourth had a tracheostomy because of respiratory obstruction and the fifth had a strongly positive Kahn test.

Significant too, is the fact that a simple palliative measure such as jejunostomy also carried a high mortality. It was presumably not possible to carry out a gastrostomy on these cases. It is clear therefore that jejunostomy serves no useful purpose as a supportive measure.

The rate of resectional surgery has risen steadily since 1952 and cases where only an exploration was done have decreased accordingly as shown in Fig. 5.

This is due to an increasing appreciation of palliative resection where growth is left behind and where a varying mass of tumour tissue is removed and continuity of the upper alimentary tract restored. This brings about a more comfortable end than that experienced from a slow and protracted starvation.

It will be seen from Fig. 5 that mortalities fell steadily between the years 1953 and 1955 as experience was gained in this field of work. There was a sharp rise, however, in the following year. This was due to a trial made at modification of the standard procedure for middle third growths. This modification consisted of a synchronous-combined abdomino-thoracic procedure which was attempted during that year. The mortality rate from this procedure was high and did not merit its continuance.

Radiotherapy has hardly been used as an elective method of treatment in Singapore. Studies from special radiotherapy centres suggest that results by these methods have been comparable with those of surgery. Radiotherapy has been available in Singapore only recently and for varying periods. Latterly it has been used as an adjunct to surgery and residual tumours have been irradiated. Recurrences in lymph nodes have responded remarkably to this form of treatment. It is too early however to assess the combined method of treatment from the point of view of an increased length of survival.

Souttar's tubes have only been used since 1954 and have steadily replaced gastrostomy as a method of palliation for inoperable cases. This method has only been used for growths in the upper and middle thirds and is a satisfactory form of palliation because it permits of feeding through normal channels. The insertion of Souttar's tubes was undertaken by the ear, nose and throat surgeons.

Operative methods selected depended on the site of the tumour.

Middle third growths were attacked by the method described by Lewis (1946) where the stomach and lower oesophagus are mobilised by the abdominal route. Resection of the oesophagus is done through a right thoracotomy, the stomach delivered into the pleural cavity through the oesophageal hiatus and continuity restored with an oesophago-gastrostomy.

Lower third growths have been resected through a left thoraco-abdominal incision after the method described by Allison (1942). In mobile growths resection

FIG. 5.—Exploration, resection and mortality rates.

is conducted after the principle of a block dissection, removing stomach, oesophagus related parts of greater and lesser omentum, spleen and left half of pancreas and all intervening tissues and lymph nodes in one piece. Continuity is restored by oesophago-gastrostomy or oesophago-jejunostomy.

Supra-aortic growths have been resected by a recently described synchronous-combined abdomino-thoraco-cervical operation done by two surgeons. The stomach is mobilized through a laparotomy. The oesophagus is mobilized through a right thoracotomy, and simultaneously delivered into the neck through a right supra-clavicular transverse incision. Continuity is then effected in the neck by oesophago-oesophagostomy after resection of most of the oesophagus (Yeoh and Cohen, 1958).

It will be noticed from Table IV that of the one hundred and seventy cases sixty-nine were resected. This is 40·6 per cent of all cases. The majority of cases were inoperable and resection was only partial.

Of the sixty-nine cases resected, nine were noted to have uncomplicated, mobile localized tumours without lymph node involvement. These growths were apparently completely excised at operation. Of these one died post-operatively giving a mortality of 11 per cent. We have failed, unfortunately, to trace four cases.

Of the remaining four, two are still alive and well after eighteen months and four and a half years. The other two died ten months and two years after operation. The former developed hoarseness of voice and the latter supraclavicular lymph nodes.

In the remaining sixty cases the growth was either incompletely removed because of fixation to surrounding vital structures or, being mobile and localized, was complicated by secondary involvement of lymph nodes which were incompletely removed. There were thirty post-operative deaths in this group, giving a mortality of 50 per cent. Only sixteen of the thirty survivals could be traced.

These survived from periods ranging from one month after discharge from hospital to fifteen months. One is still alive after twenty-two months. He developed secondaries in the supraclavicular lymph nodes. These were successfully treated with radiotherapy. He remains free from dysphagia and is in fair health.

One case who survived fifteen months developed multiple secondaries in the skin of scalp, abdominal wall and in isolated lymph nodes eight months after operation. He remained free from dysphagia.

We consider that apart from offering a possibility of cure, the mobile uncomplicated growth offers an enormously greater chance of immediate post-operative survival. This is concluded by the great difference in mortality rates in the two groups. It is felt that smooth excision of the mobile tumour as opposed to the difficult dissection of the fixed growth accounts for this.

We feel that resections should be attempted on all cases because it is impossible to tell at operation which cases are likely to survive for any length of time.

### *Post-operative Complications*

Of those who survived operation five or 13 per cent of cases suffered from post-operative complications. Three had wound infections; one developed a right hydrothorax and one a fistula in the anastomosis which responded to continuous suction drainage of the pleural cavity.

### *Causes of Post-operative Deaths*

We were able to obtain autopsies on only thirteen, or 42 per cent of cases. In the remaining cases diagnosis was clinical. The causes of death in those who underwent resection were as follows :

| Cause of death | Number of cases |
|---|---|
| Post-operative shock | 2 |
| Pneumonia | 5 |
| Pulmonary collapse | 5 |
| Pulmonary oedema | 3 |
| Gangrene stomach | 4 |
| Fistula in anastomosis | 6 |
| Empyema | 1 |
| Wound infection | 2 |
| Cardiac failure | 1 |
| Chylothorax | 1 |
| Tracheal fistula | 1 |

It is interesting to note that thirteen of the thirty-one deaths were due to lung complications. This is nearly half of all the deaths and emphasizes the need for careful pre- and post-operative physiotherapy. Lnng complications are not surprising in view of the fact that these cases are often emphysematous. We are becoming more and more inclined to do tracheostomies for direct suction of secretions at the slightest indication of pulmonary complications.

One case who died of " gangrene of stomach " on the twenty-eighth post-operative day is worthy of special mention. This patient was making smooth post-operative progress and often complained of being hungry. He collapsed and died suddenly at night. Autopsy revealed a right thoracic cavity full of food and there is little doubt that the stomach had disrupted following a large meal which had been smuggled in by the patient's relatives. There was a gangrene of the upper half of the stomach with a clear line of demarcation separating it from a healthy lower half. Doubtless the large meal had caused a severe and sudden distension of the stomach. This in turn had completely obstructed the already limited blood supply to this part of the organ. This indicates that there should be some discretion in eating for a considerable time after operation.

### SUMMARY AND CONCLUSIONS

A paper is presented reviewing one hundred and seventy cases of primary cancers of the oesophagus as seen in the Surgical Professorial Unit of the Civil General Hospital, Singapore, between the years 1948 to 1957.

It is shown that this lesion is more common among the Chinese than in the other communities living in Singapore. No reason is ascribed for this difference.

This work confirms that of others that this condition is more common in males.

This lesion is shown to occur more commonly in those of the lower income groups.

A review is made of the symptomatology and the cardinal symptoms of this disease are dysphagia, loss of weight, and regurgitation or vomiting.

It appears that dysphagia occurs as two main types, progressive and intermittent, and reasons are advanced for these two forms.

It is shown that the site of discomfort is not necessarily related to the site of obstruction.

An assessment is made of the general physical condition of these patients and various aspects of their physical states are discussed. It is pointed out that there is a paucity of physical characteristics in this disease.

Methods of special investigation and their value are discussed.

The site and pathology of this tumour are described and mention is made of the weakness of the present mode of classifying these growths as belonging to upper, middle and lower thirds of the oesophagus.

The methods by which these one hundred and seventy cases were managed are discussed. Comment is made on the steadily changing trend towards more radical procedures due to an increase of surgical resources in Singapore. A plea is made for the benefits that accrue from resectional surgery and from the re-establishment of continuity of the alimentary tract. A difference is made between operability and resectability. It is pointed out that resectability in the majority of cases can only be determined at operation and that satisfactory palliation is obtained even if tumour tissue has to be left behind. It is pointed out that resectability is not dependent on the duration of dysphagia.

The possibility of long term survivals and even cures is considered.

The operative methods used are briefly summarized and mortalities from all the procedures analysed. An enormous difference in immediate post-operative mortality rates is found between those cases where the growth is mobile and easily resected as opposed to those that are fixed and call for considerable dissection. In the former the mortality rate was 11 per cent. In the latter it was 50 per cent.

A review of such cases as have been followed up is made and a résumé is presented of their period of survival and final cause of death.

Post-operative complications and causes of death are reviewed.

We are grateful to Mr. Tye Cho Yook of the Department of Social Medicine and Public Health for his help in the statistical analyses and to Miss Fam Kim Lan for her untiring secretarial help.

### REFERENCES

AIRD, I.—(1957) 'Companion in Surgical Studies.' Edinburgh. (E. & S. Livingstone.)
ALLISON, P. R.—(1942) *Brit. J. Surg.*, **30**, 132.
BRTISH EMPIRE CANCER CAMPAIGN—(1942) *Ann. Rep.*, **19**, 65.
FRANKLIN, R. H.—(1952) 'Surgery of the Oesophagus.' London. (Edward Arnold & Co.)
KAUFMAN, E.—(1929) 'Pathology for Students and Practitioners.' Philadelphia. (Blackiston), p. 638.
LEWIS, I.—(1946) *Brit. J. Surg.*, **34**, 18.
PARKER, E. F., HANNA, C. B. AND POSTLETHWAITE, R. W.—(1952) *Ann. Surg.*, **135**, 697.
YEOH, G. S. AND COHEN, Y.—(1958) *Aust. N.Z. J. Surg.*, **28**, 18.